# Supplementary material for: A multiplex extracellular interactome screening method employing high-avidity nanoparticles
Source: bioRxiv. 2025 Jul 14:2025.07.09.663943. Preprint. [Version 1] doi: 10.1101/2025.07.09.663943 (PMC12338595; doi:10.1101/2025.07.09.663943)
Supplement: Supplement 6 [file NIHPP2025.07.09.663943v1-supplement-6.pdf]

bioRxiv preprint doi: https://doi.org/10.1101/2025.07.09.663943; this version posted July 14, 2025. The copyright holder has placed this preprint (which was not certified by peer review) in the Public Domain. It is no longer restricted by copyright. Anyone can legally share, reuse, remix, or adapt this material for any purpose without crediting the original authors.

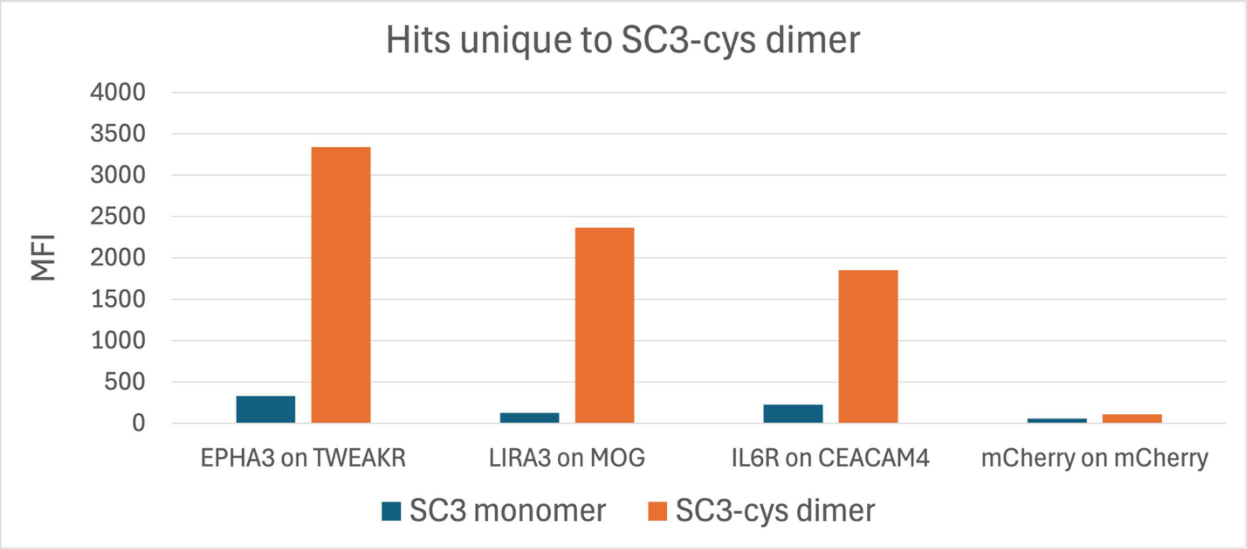

Fig. 5, supplementary figure 1

The bar graph shows MFIs for three hits in a run (M2/D1) in which preys were incubated with a bead region pool containing both monomeric SC3 and dimeric SC3 beads. We observed that these three interactions passed our statistical tests for dimeric SC3 beads, but had MFIs near background levels on monomeric SC3 beads. These are among the 15 new hits listed in Fig. 7, supplementary table 1. See Figs. 6 data supplement for raw data from these and other runs.

bioRxiv preprint doi: https://doi.org/10.1101/2025.07.09.663943; this version posted July 14, 2025. The copyright holder has placed this preprint (which was not certified by peer review) in the Public Domain. It is no longer restricted by copyright. Anyone can legally share, reuse, remix, or adapt this material for any purpose without crediting the original authors.

Figure 6S

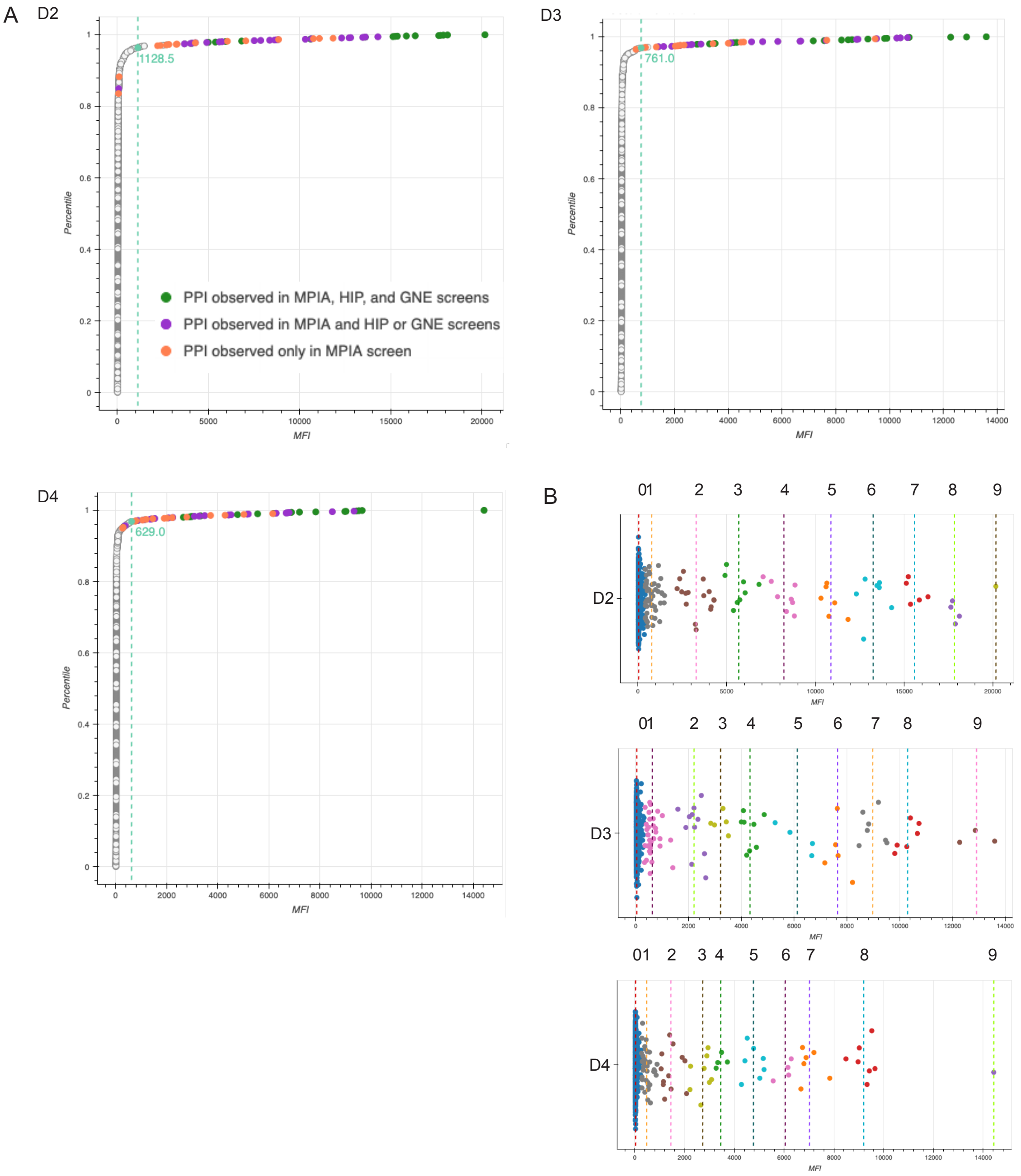

Figure 6, supplementary figure 1

ECDFs and K-means plots for runs D2-D4. See Fig. 6 legend for details on these plots.

bioRxiv preprint doi: https://doi.org/10.1101/2025.07.09.663943; this version posted July 14, 2025. The copyright holder has placed this preprint (which was not certified by peer review) in the Public Domain. It is no longer restricted by copyright. Anyone can legally share, reuse, remix, or adapt this material for any purpose without crediting the original authors.

PPIs for binding pairs not observed in HIP1 or GNE screens

Prey::Bait	# Runs Observed	Bidirectional	Run D1	Run D2	Run D3	Run D4
EDAR::ICAM5	4	yes	2726	3277	2032	2016
ICAM5::EDAR	3	yes	1671	67	2230	2787
EPHA3::LEP	4	yes	11163	10762	7666	5024
LEP::EPHA3	4	yes	12477	11838	9462	6155
MOG::ICAM5	4	yes	5329	6041	4571	1459
ICAM5::MOG	3	yes	5563	100	4000	3722
IL6R::TIGIT	4	no	5449	7052	3429	2218
EPHA3::TWEAKR	4	no	3336	3235	2140	805
IL6R::CEACAM4	4	no	1850	2830	1322	1059
IL6R::IgSF9b	4	no	8025	8849	4474	4286
LIRA3::MOG	3	no	2363	2354	920	300
IL6R::IL6R	4	homophilic	2645	4279	2121	1200
TIE1::TIE1	4	homophilic	3280	2695	2355	1906
LIRB4::LIRB4	3	homophilic	2469	2208	564	765
VSIG8::VSIG8	3	homophilic	7000	11087	918	273

PPIs in prey::bait orientation not observed in HIP1 or GNE screens

Prey::Bait	# Runs Observed	Bidirectional	Run D1	Run D2	Run D3	Run D4
LIRA3::IL6R	4	yes	10068	10313	6693	5194
PD-L2::PD-L1	4	no	4197	4013	2206	1087
IgSF9b::LIRA3	4	yes	1560	4906	2603	1402
IL6R::LIRB1	4	yes	2885	4126	1899	1536
AXL::VSIG10L	4	yes	6017	5741	4077	3086
VSIG8::VISTA	4	yes	6906	7514	1404	377
PD-L2::ICAM5	4	yes	8046	8362	4866	3270
ISLR2::LEP	4	yes	13992	13608	8823	6677
WFIKKN2::Punc E11	4	yes	14815	14294.5	10404	8484

Fig. 7, supplementary Table 1

New hits identified in MPIA screens with dimeric SC3 bait beads. MFIs are listed for each run in the last four columns. *Top*. PPIs for binding pairs that were not observed in HIP1 or GNE screens. PPIs with ICAM5 prey were not seen in run D2 because that prey was not added to the well as the result of an experimental error. *Bottom*. PPIs in a prey::bait orientation that was not observed in HIP1 or GNE screens. In the HIP1 or GNE screens, these PPIs were observed in the opposite prey::bait orientation. We also detected EPHA3::LIRA3 and EPHA3::PD-L1 as hits. In these cases, this orientation was not tested in either screen, but the reverse orientation was observed as a hit in GNE.
